# Effect of omega‐3 fatty acids supplementation on indirect blood markers of exercise‐induced muscle damage: Systematic review and meta‐analysis of randomized controlled trials

**DOI:** 10.1002/fsn3.2598

**Published:** 2021-09-21

**Authors:** Gao Xin, Hesam Eshaghi

**Affiliations:** ^1^ School of Physical Education University of South China Hengyang China; ^2^ Department of Community Nutrition School of Nutritional Sciences and Dietetics Tehran University of Medical Sciences Tehran Iran

**Keywords:** creatine kinase, lactate dehydrogenase, meta‐analysis, muscle damage, myoglobin, omega 3 fat

## Abstract

**Background:**

Omega 3 fatty acids supplementation may have an attenuative effect on exercise‐induced muscle damage (EIMD) through the cell membrane stabilization. The purpose of the present meta‐analysis was to evaluate the effects of omega 3 fatty acids supplementation, on indirect blood markers of muscle damage following EIMD in trained and untrained individuals.

**Methods:**

Scopus, Medline, and Google scholar systematically searched up to January 2021. The Cochrane Collaboration tool was used for the quality of studies. Random‐effects model, weighted mean difference (WMD), and 95% confidence interval (CI) were applied for the overall effect estimating. The heterogeneity between studies was evaluated applying the chi‐squared and I^2^ statistic.

**Results:**

The outcomes showed a significant effect of omega 3 supplementation on reducing creatine kinase (CK), lactate dehydrogenase (LDH), and myoglobin (Mb) concentrations. In addition, a subgroup analysis indicated a significant reduction in CK, LDH, and Mb concentrations, based on follow‐ups after exercise, studies duration, time of supplementation, and training status.

**Conclusion:**

The current meta‐analysis indicated an efficacy of omega 3 in reducing CK, LDH, and Mb serum concentration among healthy individuals, overall and in subgroups analysis. Thus, omega 3 should be considered as a priority EIMD recovery agent in interventions.

## Graphical abstract

The current meta‐analysis indicated an efficacy of omega 3 in reducing CK, LDH, and Mb serum concentration among healthy individuals, overall, and in subgroups analysis, based on follow‐ups after exercise, omega 3 doses, studies duration, time of supplementation, exercise type, and training status.

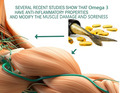

## 1 INTRODUCTION

Damaged muscle cells start a reactions cascade that leads to a complicated and prolonged interaction between protein synthesis and degradation (Shen et al., 2018). Therewith, while protein turnover is increased, generally degradation exceeds synthesis, since the breakdown of protein results, leading to muscle atrophy, muscle degeneration, and exercise‐induced muscle damage (EIMD; Damas et al., 2016). Exercise‐induced muscle damage lead to alteration in muscle protein structure, reductions in muscle strength, and impaired muscle function (King & Baker, 2020). Exercise‐induced muscle damage is associated with morphological changes, increases in serum skeletal muscle enzymes (creatine kinase (CK), lactate dehydrogenase (LDH), and myoglobin (Mb) concentrations), and decrements of force production and neuromuscular deficits. This EIMD manifests as a reduced range of motion, decrease in neuromuscular function, and limb swelling. These symptoms impair muscle function and inhibit the potential to engage in high‐intensity exercise on subsequent days, which is often required by athletic populations (Mohamad‐Panahi et al., 2013).

In exercise process, recovery after physical activity is of uttermost importance. The recovery procedure attenuates the negative outcomes of EIMD and DOMS and enhances muscle function in physical activities (Rahimi et al., 2018). The recovery importance gets even more crucial when the training level elevates, for the sake of the duration and frequency of exercise, that are very high in trained athletes (Owens et al., 2019). Several conditions of exercise, such as eccentric training or long‐duration exercise bouts, may also produce specific recovery matters, because they produce a heavy physiological pressure on the motor units. For example, it has been shown that exercise can cause muscle injury (Mielgo‐Ayuso et al., 2020), which operates an inflammatory reaction. This reaction is related to a slow muscle recovery, that can last some days (Delecroix et al., 2017). Thus, various protocols have been planed to accelerate recovery after exercise. These protocols may include physical therapies (e.g., compression and water immersion), manual therapies (e.g., stretching and massage), and nutritional therapies (Bongiovanni et al., 2020). In relevance to nutritional therapies, some supplements with a potential recovery improving effect have been examined: vitamins (Stepanyan et al., 2014), antioxidants (Delecroix et al., 2017; Fang & Nasir, 2020), carbohydrates (Stearns et al., 2010), proteins (Stearns et al., 2010), branched‐chain amino acids (BCAA; Rahimi et al., 2017) and beta‐hydroxy beta‐methylbutyric acid (HMB; Rahimi et al., 2018).

One category of supplements that shows to have anti‐inflammatory features is omega 3 long‐chain polyunsaturated fatty acids (LC‐PUFA), such as docosahexaenoic acid (DHA; 22:6 n3) and eicosapentaeoic acid (EPA;20:5 n3), found in fish oil (Oppedisano et al., 2020). The PUFAs, especially EPA and DHA, have been suggested to become incorporated into the cellular membranes (Xiao et al., 2020). This procedure can modify the release of muscle enzymes and the more pro‐inflammatory 2 series prostaglandins, thromboxanes, and prostacyclins (Christie & Harwood, 2020; Das, 2005).

Various studies have suggested that omega 3 LC‐PUFA have beneficial effects in human health and many inflammatory diseases (Schunck et al., 2018) and may operate as important energetic molecules in exercise that can modulate oxidative stress and inflammatory responses (Tocher, 2015). The mechanism for anti‐inflammatory features of omega 3 LC‐PUFA comprises membrane‐derived omega‐6 fats with omega 3 fats substrate competition for lipoxygenase and cyclooxygenase (COX) enzymes causing less inflammatory eicosanoids production and decreased generation of inflammatory eicosanoids (Calder, 2012; Wall et al., 2010). Moreover, omega 3 LC‐PUFA have effects as ligands in nuclear for nuclear factor kappa B (NF‐KB) and peroxisome proliferator‐activated receptors, therefore influencing inflammatory factors transcription such as adhesion molecules and cytokines (Iverson et al., 2018; Schunck et al., 2018).

Numerous studies have sought to assess whether supplementation of omega 3 fatty acids can decrease the muscle damage degree, oxidative stress, and inflammation after exercise (Arab‐Tehrany et al., 2012; Davinelli et al., 2019; Tan & Norhaizan, 2019). However, more investigations have illustrated a significant effect of omega 3 LC‐PUFA in relation to improving EIMD, DOMS, oxidative stress, and inflammation following exercise (Gray et al., 2014; Jouris et al., 2011; Rajabi et al., 2013; Santos et al., 2012; Tartibian et al., 2011), and some studies have represented no effect (Lenn et al., 2002; López‐Seoane et al., 2021; Phillips et al., 2003). It is possible that the differences in exercise protocols, supplementation duration and dosage, participant's population, timing of supplementation and measurements and biomarkers selection relate to the discrepancies in the outcomes between investigations. Thus, the main aim of the current review was to evaluate whether omega 3 fatty acids supplementation with different doses and during the days before and after different exercise protocols accelerates recovery of EIMD and attenuates the rise in circulating plasma markers of muscle damage. The present meta‐analysis evaluated EIMD indirect markers, including CK, LDH, and Mb among trained and untrained healthy subjects of both genders.

## 2 METHODS

### 2.1 Strategy of search

Current review study was presented according to the guidelines of Preferred Reporting Items for Systematic Reviews and Meta‐Analyses (PRISMA; Liberati et al., 2009a). A computerized search was carried out from inception to January 2021 applying diverse databases including Scopus, PubMed, ISI Web of Science, and a supplementary search in Google Scholar. The literature search was restricted to English articles. The following MeSH and non‐MeSH terms and their combinations were used, including: “fatty acids, omega 3,” “omega 3,” “n‐3 polyunsaturated fatty acid,” “n‐3 PUFA,” “docosahexaenoic acid,” “eicosapentaenoic acid,” “EPA,” “DHA,” “exercise,” “physical exercise,” “eccentric exercise,” “aerobic exercise,” “athlete,” “muscle soreness,” “muscle damage,” “creatine kinase,” “lactate dehydrogenase,” “myoglobin,” “muscle enzyme activity,” “controlled trial,” “random,” “randomly,” “randomized clinical trial,” “randomized,” “randomised,” “RCT,” “blinded,” “double blind,” “double blinded,” “trial,” “controlled clinical trial,” “crossover procedure,” “cross‐over trial,” “double blind procedure,” and “equivalence trial.” Reference lists of all articles were screened for articles that are more eligible.

### 2.2 Criteria for eligibility

Articles were selected according to the Population–Intervention–Comparator–Outcomes–Study design (PICOS) (Liberati et al., 2009a), including The Population (healthy participants aged more than eighteen years old without muscles damage or injury history), Intervention (omega 3 supplementation), Comparison (matched control group), and Outcome (EIMD indices including CK, LDH, and Mb concentration), that were performed in study design of randomized controlled trials (RCTs).

All RCTs were included in the current meta‐analysis if met our inclusion criteria: (1) original researches in RCT study design; (2) participants received oral omega 3 supplementation, as a nutritional strategy; (3) presented at least one muscle damage indices (CK, LDH, and Mb) measurement; and (4) reporting interest data as mean and standard deviation (*SD*) of CK, LDH, and Mb in supplementation and placebo both groups. Exclusion criteria were as follows: (1) consuming omega 3 mixture in supplementation group only, not including a placebo group; (2) animal studies; (3) trials without control groups, nonrandomized, or semi‐experimental trials; (4) case reports, editorial articles, or letters to the editor; and (5) duplicate articles with same participant.

### 2.3 Strategy of selection

Following initial search, all papers recorded in manual searches or electronic searches were entered into EndNote software for checking (EndNote X6; Thomson Reuters, New York). According to search strategy, titles and abstracts of papers were screened. Papers were assessed independently by two authors and selected based on the inclusion criteria. Papers including eligibility criteria in the title and abstract checking were selected to be evaluated by full text. If our inclusion criteria were met, all of RCTs were included in current meta‐analysis. We applied a pre‐design form to select papers eligible for inclusion in the review, according to the data within the full text. Contradictions between the reviewer authors were dissolved by the third researcher or consensus.

### 2.4 Extraction of data

Two independent reviewers extracted interest data applying a standardized electronic form (Excel and Microsoft Office) including first author's name, country and year of publication, design of research, sample size, age and gender of participants, duration of intervention, and dose of omega 3. In addition, authors extracted baseline and after the intervention mean and *SD* of interest data (CK, LDH, and Mb). Any presented standard errors (SE) of mean were changed to SDs via this formula: (*SD* = *SEM* × √*n* (*n* is the subjects’ number in intervention and placebo groups). Finally, in papers that depicted data in figures, extraction of data was carried out applying Graph Digitizer 2.24 software (Fedorov, 2002).

### 2.5 Quality of studies

As regards, it has been indicated that inclusion of high risk of bias RCTs may distort the results of a meta‐analysis study (Higgins et al., 2011; Liberati et al., 2009b), and the Cochrane Collaboration tool was used for measuring the risk of bias. All the included RCTs quality were assessed by these items: randomization sequence generation; allocation concealment; blinding of participants, personnel, investigator, and assessor; and attrition rates. Mentioned items were given a rating of low, unclear, or high risk of bias. A study was ranked low‐, medium‐, or high‐risk bias overall, according to the key items of participants and assessor blinding, allocation concealment, and reporting of attrition rates (Low = Low risk of bias for all key items, Medium = Low or unclear risk of bias for all key items, and High = High risk of bias for one or more key items; Higgins et al., 2011).

### 2.6 Analysis and treatment effect measures

Mean differences and *SD* were computed for continuous measures for every trial. Standardized mean changes were used for variables pooled on the different scales. For papers with no mean change *SD*, this formula was applied: *SD* change = square root [(*SD* final^2^ + *SD* baseline^2^) − (2 × 0.8 × *SD* final × *SD* baseline)] (Borenstein et al., 2011). Heterogeneity of studies was evaluated applying the chi‐squared (χ^2^) test and quantified by the *I* ^2^ statistic, which reports the percentage of the total variation across trials that are attributable to heterogeneity rather than to chance. *p*‐value of <.05 was defined as significant heterogeneity.

For estimating the overall effect, the weighted mean differences (WMDs) with 95% confidence intervals (CIs) were calculated using the random‐effects model. To evaluate whether the outcomes could have been influenced by a single study distinctly, a sensitivity analysis was carried out (Tobias, 1999). Also, subgroup analysis was performed, according to follow‐up times measurements after exercise (immediately, 24, 48, 72, and 96 h after exercise), duration of trials (acute (single dose), lower than 1 month and more than 1 month), time of supplementation (before exercise, after exercise, and before and after exercise), and training status (trained and untrained). Furthermore, Egger's regression asymmetry and test Begg's rank correlation test were used to evaluate publication bias. The effect sizes versus their corresponding SE (differences in means) were depicted by funnel plots. Moreover, statistical analyses were conducted applying STATA 11.2 software (StataCorp).

## 3 RESULTS

### 3.1 Findings from search and included studies overview

We found 254 related papers in our search. After removing duplicates, an extensive titles and abstracts screening was conducted on 251 papers. After checking the inclusion and exclusion criteria for the eligibility, 20 papers remained. At last, 10 articles, including 36 effect sizes for CK concentration, 9 effect sizes for LDH concentration, and 11 effect sizes for Mb concentration were identified in the present meta‐analysis, which investigated a total of 239, 105, and 86 subjects respectively. The numbers is inclusive of subjects who were dropouts in some studies. All subjects tended to be young aged 18.2–23.5 years. Furthermore, all subjects were men, except in one study that both genders participated (*n* = 10) (Lenn et al., 2002).

**FIGURE 1:**
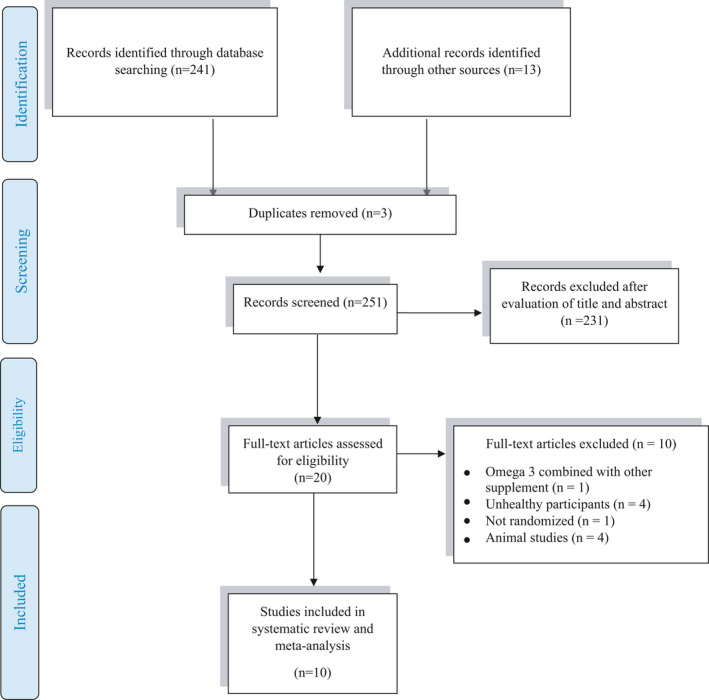
Preferred Reporting Items for Systematic Reviews and Meta‐Analyses (PRISMA) flow diagram of study selection process

The selection procedure and reasons for excluding the studies presented in Figure 1 and Table 1 indicate the basic characteristics of the studies in our review. Briefly, the papers were published between 2002 and 2017. The total subjects’ number who completed the trials in inclusion criteria was 122 subjects in the supplement and 117 in the placebo groups for CK concentration, 51subjects in the supplement and 54 in the placebo groups for LDH concentration, and 43 subjects in the supplement, and 43 in the placebo groups for Mb concentration. The dose of fish oil, mussel oil, algal species, and purslane extract supplementation was 0.8 to 3 g/day among these studies, and the duration of them ranged between 1 day and 62 days. All studies used a randomized placebo‐controlled fashion design except one study (Mickleborough et al., 2015) that used randomized crossover design. The effect of omega 3 on CK, LDH, and Mb together was examined in one study (Tartibian et al., 2011), and 4 studies reported only CK concentration (DiLorenzo et al., 2014; Gray et al., 2014; Jakeman et al., 2017; Lenn et al., 2002).

**TABLE 1:** Characteristics of the included studies

Author (year)	Study Design Characteristics								Average age (years)	Sample Size		Outcomes
	Design	country	training status	Omega 3 dose (g/day)	Omega 3 type	Duration (days)	Consumption time	Gender		Omega 3	Control	
Jakeman et al. (2017)	RP	UK	T	3 (EPA 750 mg, DHA 50 mg)	fish oil	1	A. Ex	M	26	9	9	CK
Jakeman et al. (2017)	RP	UK	T	3 (EPA 150 mg, DHA 100 mg)	fish oil	1	A. Ex	M	26	9	9	CK
Tsuchiya et al. (2016)	RP	Japan	U	2.4 (300 mg EPA, 130 mg DHA)	fish oil	62	B. Ex, A. Ex	M	19.5	12	12	CK, Mb
Mickleborough et al. (2015)	CP	USA	U	0.8 (58 mg EPA, 44 mg DHA)	mussel oil blend	30	B. Ex, A. Ex	M	22	16	16	CK, Mb
Gray et al. (2014)	RP	UK	U	3 (1,300 mg EPA, 300 mg DHA)	fish oil	42	B. Ex	M	23	10	10	CK
DiLorenzo et al. (2014)	RP	USA	U	2 (500 mg DHA)	algal species	28	B. Ex	M	21.8	10	11	CK
Rajabi et al. (2013)	RP	Iran	U	2 (^)	fish oil	30	B. Ex, A. Ex	M	20.5	10	10	CK, LDH
Meamarbashi and Abedini (2011)	RP	Iran	U	1.2 (^)	purslane extract	5	B. Ex, A. Ex	M	18.2	10	10	CK, LDH
Tartibian et al. (2011)	RP	Iran	U	1.8(324 mg EPA, 216 mg of DHA)	fish oil	30	B. Ex	M	29.7	15	15	CK, LDH, Mb
Phillips et al. (2003)	RP	USA	U	0.8 (200‐mg DHA)	fish oil	14	B. Ex, A. Ex	M	22.1	16	19	CK, LDH
Lenn et al. (2002)	RP	USA	U	1.8 (^)	fish oil	30	B. Ex	M & F	23.5	5	5	CK

Abbreviations: A, aerobic training; A. Ex, after exercise; B. Ex, before exercise; CK, creatine kinase; CP, crossover study; D, Days; F, Female; LDH, lactate dehydrogenase; M, male; Mb, myoglobin R, resistance training; RP, randomized controlled clinical trial; T, trained; ^, unspecified or unknown; U, untrained; Y, years.

Most of the studies measured several follow‐up times for each index (immediately, 1, 2, 3, 24, 48, 72 and 96 h after exercise). We concentrated on results presented immediately after exercise and subsequent hours (24, 48, 72, and 96 h after exercise). Five effect sizes in five articles had immediately after exercise (Gray et al., 2014; Lenn et al., 2002; Meamarbashi & Abedini, 2011; Mickleborough et al., 2015; Tsuchiya et al., 2016); Nine effect sizes in eight articles presented 24 h (Gray et al., 2014; Jakeman et al., 2017; Lenn et al., 2002; Meamarbashi & Abedini, 2011; Mickleborough et al., 2015; Rajabi et al., 2013; Tartibian et al., 2011; Tsuchiya et al., 2016); Ten effect sizes in nine articles had 48 h (DiLorenzo et al., 2014; Gray et al., 2014; Jakeman et al., 2017; Lenn et al., 2002; Meamarbashi & Abedini, 2011; Mickleborough et al., 2015; Rajabi et al., 2013; Tartibian et al., 2011; Tsuchiya et al., 2016); Eight effect sizes in seven articles presented 72 h (Gray et al., 2014; Jakeman et al., 2017; Lenn et al., 2002; Mickleborough et al., 2015; Phillips et al., 2003; Rajabi et al., 2013; Tsuchiya et al., 2016), and Four effect sizes in three articles presented 96 h (DiLorenzo et al., 2014; Jakeman et al., 2017; Mickleborough et al., 2015).

Also, the timing when omega 3 supplement must be consumed is debatable. Eighteen effect sizes in 6 studies had before and after exercise supplementation (Meamarbashi & Abedini, 2011; Mickleborough et al., 2015; Phillips et al., 2003; Rajabi et al., 2013; Tartibian et al., 2011; Tsuchiya et al., 2016), Ten effect sizes in 3 studies had before exercise supplementation (DiLorenzo et al., 2014; Gray et al., 2014; Lenn et al., 2002). Just Jakeman et al. investigated the effects of the timing of supplement ingestion on muscle damage after exercise (Jakeman et al., 2017).

### 3.2 Quality assessments outcomes

The quality details of bias assessment are indicated in Table 2. In brief, participants’ random allocation was illustrated in all included studies. Nevertheless, two articles mentioned the random sequence generation method and reported allocation concealment (Jakeman et al., 2017; Tsuchiya et al., 2016). All articles indicated low risk of bias according to incomplete outcome. For selective outcome reporting, most of the articles had a low risk of bias although one study represented high risk of bias (Lenn et al., 2002) and three studies represented unclear risk of bias (Phillips et al., 2003; Rajabi et al., 2013; Tartibian et al., 2011) according to selective reporting. Moreover, all articles had a unclear or high risk of bias for participants and personnel blinding and outcome assessors blinding except two studies that indicated low‐risk regarding participants, personnel, and outcome assessment blinding (Jakeman et al., 2017; Tsuchiya et al., 2016). Most articles reported low risk of bias about other potential threats to validity including a potential source of bias related to the particular study design applied; or had some problem like study has been claimed to have been fraudulent. Finally, most of the articles had medium overall risk of bias, two articles had low overall risk of bias (Jakeman et al., 2017; Tsuchiya et al., 2016), and two articles had high overall risk of bias (DiLorenzo et al., 2014; Lenn et al., 2002).

**TABLE 2:** Cochrane risk of bias assessment

Study	Random sequence generation	Allocation concealment	Blinding of participants and personnel	Blinding of outcome assessment	Incomplete outcome data	Selective outcome reporting	Other sources of bias	Overall risk of bias
Jakeman et al. (2017)	L	L	L	L	L	L	L	Low
Tsuchiya et al. (2016)	L	L	L	L	L	L	L	Low
Mickleborough et al. (2015)	U	U	U	U	L	L	L	Medium
Gray et al. (2014)	U	U	U	U	L	L	L	Medium
DiLorenzo et al. (2014)	U	U	H	H	L	L	U	High
Rajabi et al. (2013)	U	U	U	U	L	U	L	Medium
Meamarbashi and Abedini (2011)	U	U	U	U	L	L	L	Medium
Tartibian et al. (2011)	U	U	U	U	L	U	L	Medium
Phillips et al. (2003)	U	U	U	U	L	U	L	Medium
Lenn et al. (2002)	U	U	H	H	L	U	L	High

Abbreviations: H, high risk of bias; L, low risk of bias; M, medium risk of bias; U, unclear risk of bias.

### 3.3 Findings from omega 3 supplementation effects on muscle damage indices meta‐analysis

#### 3.3.1 Omega 3 supplementation effect on CK concentration

**FIGURE 2:**
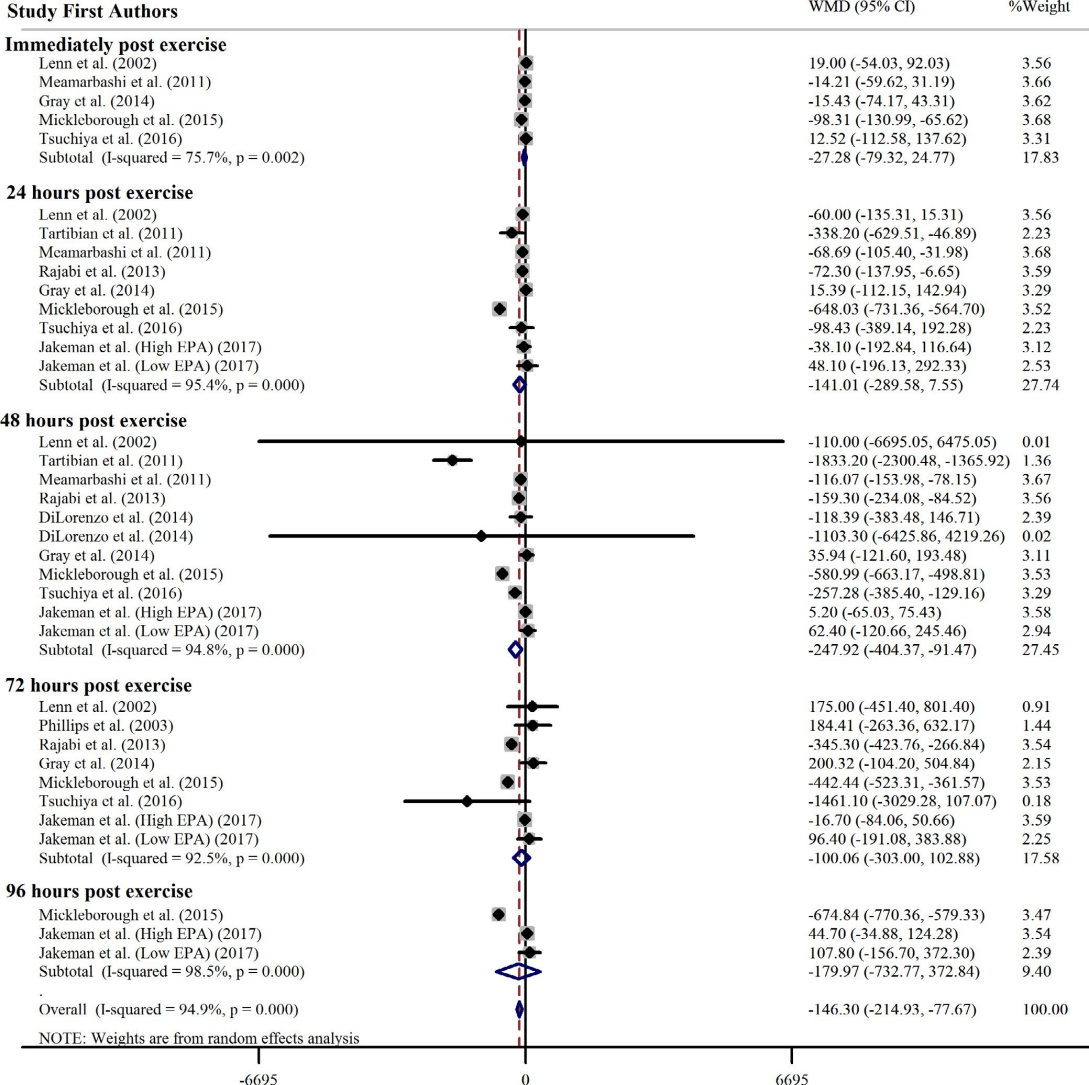
Forest plot of the effect of omega 3 supplementation on CK subgrouped by follow up times after exercise. CI, confidence interval; WMD, weighted mean difference

According to analysis on 36 effect sizes, in overall, omega 3 supplementation decreases CK concentration significantly: (WMD = −146.30 IU L^−1^, 95% CI: −214.93, −77.67; *p* <.001). Significant heterogeneity observed among the articles (*p* =.000, *I* ^2^ = 94.9%) (Figure 2). For assessing whether the omega 3 supplementation effect on serum CK concentration is different according to subgroups, meta‐analysis was carried out based on follow‐ups after exercise, studies duration, time of supplementation, and training status (Table 3). Subgroup analysis showed that omega 3 consumption has a significant reduction effect on CK concentrations in RCTs with 48 h follow‐ups after exercise, lower and more than 1 month (acute supplementation was not significant), RCTs with before and after exercise time of supplementation, and RCTs on untrained participant.

**TABLE 3:** Subgroup analysis to assess the effect of omega 3 on CK concentration

Subgrouped by	No. of effect sizes	WMD^a^	95% CI	*p* Value	*I* ^2^ (%)
Follow‐ups after exercise					
Immediately	5	−27.276	−79.319, 24.768	0.304	75.7
24 h	9	−141.013	−289.578, 7.552	0.063	95.4
48 h	11	−247.920	−404.367, −91.472	**0.002** ^*^	94.8
72 h	8	−100.063	−303.002, 102.875	0.334	92.5
96 h	3	−179.965	−732.766, 372.836	0.523	98.5
Duration					
Acute (single dose in 1 day)	8	11.809	−26.100, 49.719	0.542	0.0
<1 month	6	−66.387	−116.186, −16.588	**0.009** ^*^	96.3
1 month ≤	22	−245.784	−355.573, −135.996	**<0.001** ^*^	61.4
Time of supplementation					
Before exercise	10	−10.490	−46.227, 25.247	0.565	0.0
After exercise	8	11.809	−26.100, 49.719	0.542	0.0
Before and after exercise	18	−296.497	−398.749, −194.245	**<0.001** ^*^	97.0
Train status					
Trained	8	11.809	−26.100, 49.719	0.542	0.0
Untrained	28	−203.143	−284.519, −121.767	**<0.001** ^*^	95.7

Abbreviation: CI, confidence interval.

^a^ Weighted mean difference calculated by random‐effects model.

* *p* < 0.05.

#### 3.3.2 Omega 3 supplementation effect on LDH concentration

**FIGURE 3:**
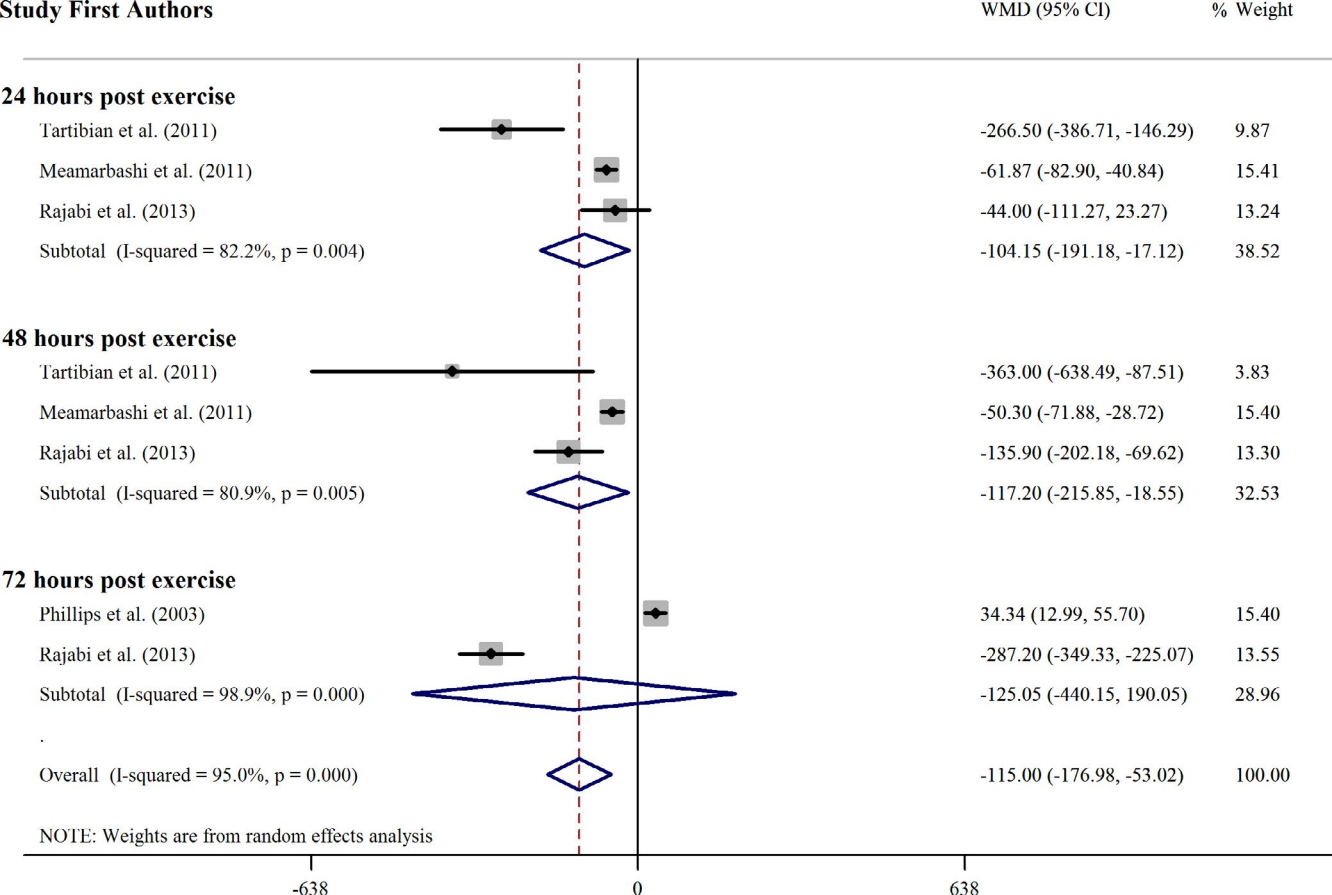
Forest plot of the effect of omega 3 supplementation on LDH subgrouped by follow up times after exercise. CI, confidence interval; WMD, weighted mean difference

Omega 3 supplementation effect of the on LDH concentration was assessed in 9 effect sizes, and analysis revealed a significant change in LDH concentration in pooled mean difference from inverse variance method (WMD = −96.20 IU L^−1^; 95% CI: −148.07, −44.32; *p* <.001). Also, significant heterogeneity observed among the articles (*p* =.000, *I* ^2^ = 94.7%) (Figure 3). To evaluate whether the omega 3 supplementation effect on LDH concentration is different according to subgroups, meta‐analysis was performed based on follow‐ups after exercise and studies duration (Table 4). Time of supplementation in studies that reported LDH was before and after exercise and all of the participants were untrained. Several subgroup analysis indicated that supplementation with omega 3 has a significant reducing effect in LDH concentrations in RCTs with 24 and 48 h follow‐ups of LDH after exercise and more than 1‐month trials.

**TABLE 4:** Subgroup analysis to assess the effect of omega 3 on LDH concentration

Subgrouped by	No. of effect sizes	WMD^a^	95% CI	*p* Value	*I* ^2^ (%)
Follow‐ups after exercise					
24 h	3	−104.152	−191.181, −17.124	**0.019** ^*^	82.2
48 h	3	−117.203	−215.852, −18.553	**0.020** ^*^	80.9
72 h	2	−125.050	−440.146, 190.046	0.437	98.9
Duration					
<1 month	4	−19.381	−63.782, 25.020	0.392	94.1
1 month ≤	5	−198.762	−311.243, −86.281	**0.001** ^*^	87.7

Abbreviation: CI, confidence interval.

^a^ Weighted mean difference calculated by random‐effects model.

* *p* < 0.05.

#### 3.3.3 Omega 3 supplementation effect on Mb concentration

**FIGURE 4:**
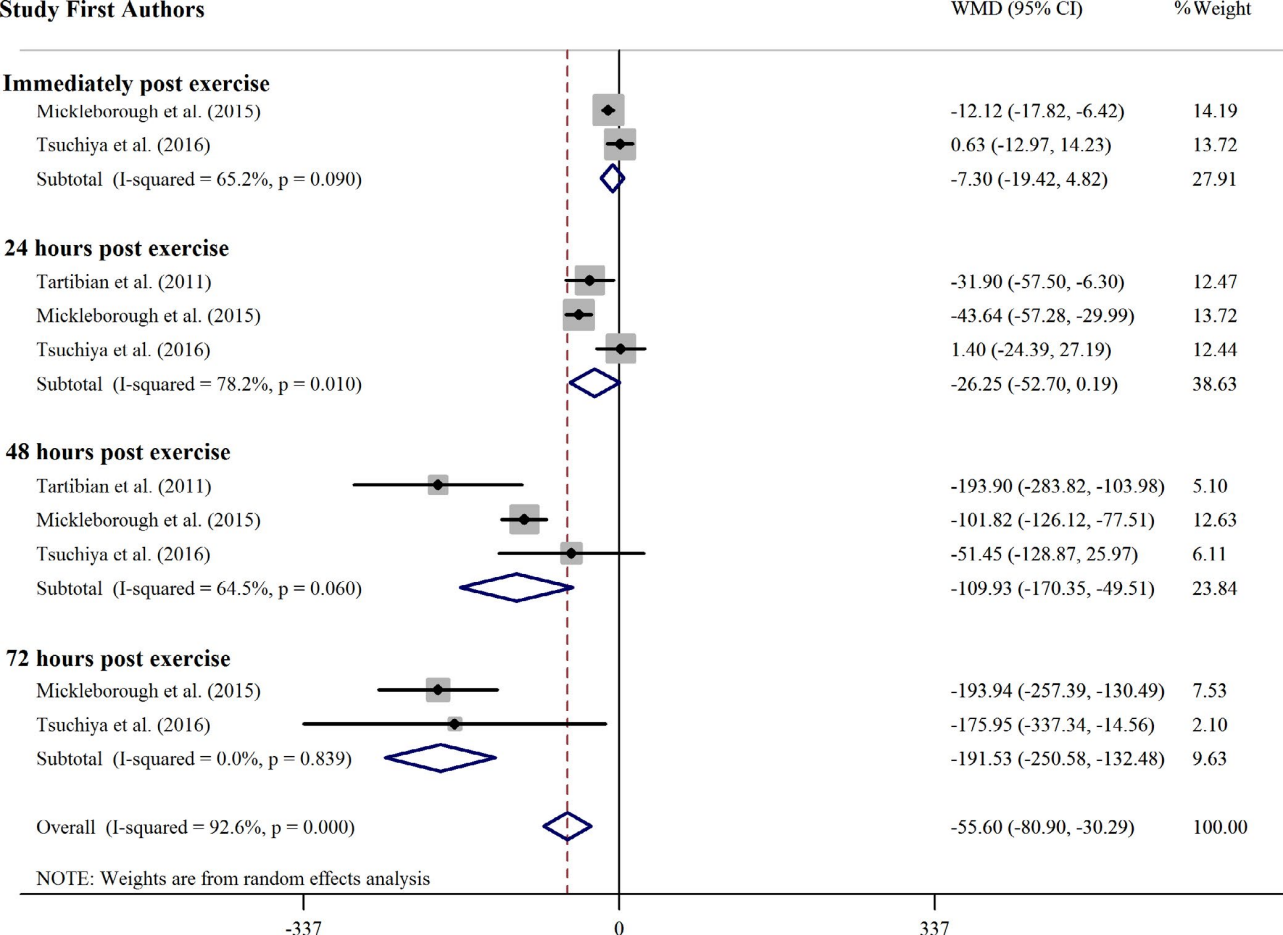
Forest plot of the effect of omega 3 supplementation on Mb subgrouped by follow up times after exercise. CI, confidence interval; WMD, weighted mean difference

The omega 3 supplementation effect on Mb concentration was assessed in 11 effect sizes and represented significant change in Mb concentration (WMD = −61.49 ng ml^−1^; 95% CI: −86.63, −36.35; *p* <.001). Also, significant heterogeneity among the articles was observed (*p* =.000, *I* ^2^ = 92.5%) (Figure 4). Subgroup analysis was conducted to evaluate whether the omega 3 supplementation effect on Mb concentration is different based on follow‐ups after exercise (Table 5). Duration of supplementation in all studies that reported Mb was more than one month, time of supplementation was before and after exercise, and all of the participants were untrained. Several subgroup analysis showed that omega 3 supplementation leads to significant decreased Mb concentrations in RCTs with 48 and 72 h measurement of Mb after exercise.

**TABLE 5:** Subgroup analysis to assess the effect of omega 3 on Mb concentration

Subgrouped by	No. of effect sizes	WMD^a^	95% CI	*p* Value	*I* ^2^ (%)
Follow‐ups after exercise					
Immediately	2	−7.302	−19.420, 4.816	0.238	65.2
24 h	3	−26.255	−52.704, 0.195	0.052	78.2
48 h	3	−109.935	−170.354, −49.515	**<0.001** ^*^	64.5
72 h	2	−191.531	−250.578, −132.484	**<0.001** ^*^	0.0

Abbreviation: CI, confidence interval.

^a^ Weighted mean difference calculated by random‐effects model.

* *p* < 0.05.

#### 3.3.4 Publication bias and Sensitivity analysis

**FIGURE 5:**
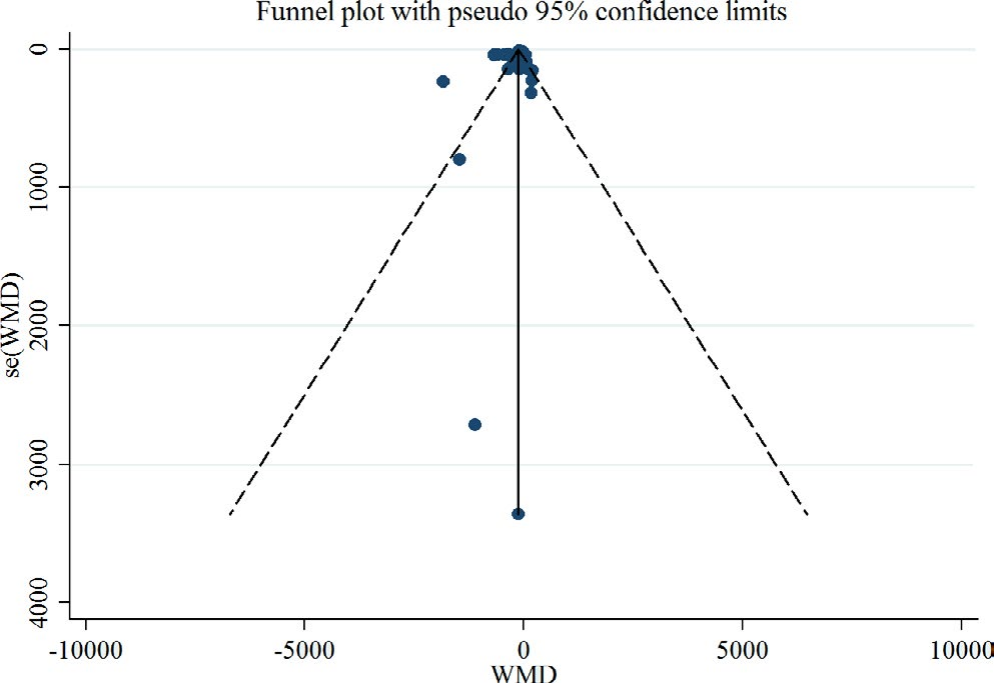
Funnel plot for evaluating publication bias in CK

Any of the studies removal from the meta‐analysis creates no alteration in the outcomes of the meta‐analysis on serum CK concentration based on sensitivity analysis, whereas the results on LDH and Mb concentration were sensitive to omitting Tartibian et al. study (Tartibian et al., 2011). Funnel plots for CK concentration were visually symmetrical (Figure 5). The Begg's test outcomes did not determine any publication bias evidence in articles that investigate the effect of omega 3 supplementation on CK concentration (Begg's test, *p* =.712). Also, the Egger's test outcomes did not determine any publication bias evidence in articles that evaluate the effect of omega 3 supplementation on LDH and Mb concentration (Egger's test, *p* =.158 and Egger's test, *p* =.198, respectively).

## 4 DISCUSSION

The aim of this review was to assess whether omega 3 consumption affected the response of muscle damage indicators to physical activity followed by the start of a training program. The current meta‐analysis results, conducted on 10 RCTs, displayed beneficial effects of omega 3 supplementation in reduction in EIMD indicators during exercise protocols of various periods.

Omega 3 fatty acids have anti‐inflammatory features for clinical situations such as asthma, arthritis, and Crohn's disease (Wall et al., 2010). In this regard, few papers have evaluated the anti‐inflammatory features of this nutrient in exercise, especially those performing anaerobic and aerobic exercise (Dalle et al., 2021; Kyriakidou et al., 2021). Despite the contradictions, most of these studies suggested omega 3 consumption attenuate the magnitude of the serum muscle enzymes response to damaging exercise, lower inflammatory response, and less myofibrillar disturbance (Mickleborough et al., 2015; Tartibian et al., 2011; Tsuchiya et al., 2016).

Previous studies suggest that higher cellular omega 3 fatty acids content decreases inflammatory factors generation through nuclear binding of PPAR and NF‐KB substrate and through competition for COX enzymes (Echeverría et al., 2019; Tapia et al., 2014). Also, the increase in serum CK concentration seen by Brenner et al. (Gil‐Quintana et al., 2020) did not parallel with the inflammatory factors, which suggests further mechanisms involved in the release of muscle enzymes. The dissociation between serum muscle enzymes and inflammatory factors responses has been indicated that omega 3 reduced serum CK concentration through the cell membrane stability alteration, which allowing decreased leakage of CK without effect on disruption of sarcomere (Norris et al., 2018). Since docosahexaenoic acid (DHA) and eicosapentaenoic acid (EPA) are incorporated into phospholipids of cell membrane, omega 3 inhibit CK leakage and other muscle damage indicators from cell membranes in peak hours after exercise with effects on fluidity of membrane (Hishikawa et al., 2017; Mason et al., 2016). So, it has been suggested that omega 3 has different physiological features, such as protective effect on membrane and anti‐inflammatory efficacy involved in inflammation and muscle proteolysis (Dyall, 2015). We suggest this mechanism for the CK, LDH, and Mb attenuation with omega 3 consumption in overall analysis, which omega 3 may have to improve muscle cells membrane stability and therewith lowered release of enzymes.

In the present meta‐analysis, subgroup analysis indicated the effect of omega 3 consumption on attenuating serum CK, LDH, and Mb is significant according to follow‐up times in 48 h after exercise. Also, this effect was significant in 24 h after exercise for LDH and in 72 h after exercise for Mb. Usually, muscle enzyme responses after exercise tend to be somewhat more delayed and peaks 1 and 2 days after exercise (Howatson et al., 2007). This phenomenon may owing to lipid peroxidation that result in membrane permeability and allows muscle enzymes such as CK, LDH, and Mb to escape (Owens et al., 2019). The protective effects on membrane and inflammation inhibitory effects of omega 3 (Adeyemi & Olayaki, 2018) may have prevented CK leakage and other enzymes from membranes of muscle cell more efficiently in peak follow‐ups after exercise and as a result less augment in CK, LDH, and Mb serum levels after 24, 48, and 72 h after exercise compared with placebo group.

Moreover, lower serum CK levels might depend on when the early site of EIMD happened, exercise type and the training status of the participants (Maughan & Gleeson, 2010), and hence the limitation of myocellular specific proteins leakage. In this regard, subgroup analysis indicated that RCTs with untrained subjects had a considerable reduction in CK concentrations with omega 3 supplementation. Thus, omega 3 supplementation is more effective for untrained participants.

Also, omega 3 supplementation more than one month decreased CK and LDH concentrations significantly. Nevertheless, acute supplementation (single dose in 1 day) had no significant effect on CK concentrations. The relationship between the duration of omega 3 supplementation and recovery from muscle damage is complex and controversial. The balance between beneficial and damaging effects of the duration of omega 3 supplementation may depend on magnitude of the inflammatory stimulus (i.e., damage), the duration of the elevation of the inflammatory factors, and the subject population (Oppedisano et al., 2020).

For example, Phillips et al. (Phillips et al., 2003) observed no effect of 7 days of ingestion of a multi‐ingredient dietary supplement with 800 mg of DHA on markers of muscle damage after eccentric exercise. In another study, the same duration of supplementation with a larger dose of omega 3 fat (3 g/day) attenuated the muscle damage markers in subjects 48 hr after performing eccentric biceps extensions (Jouris et al., 2011). Lenn et al. (2002) used a longer supplementation period of 30 days with a dose of omega 3 fats similar to DiLorenzo et al. (2014) before the eccentric exercise bout with significant effect on markers of muscle damage. On the contrary, in Jakeman et al. study (Jakeman et al., 2017), an acute dose of n‐3 PUFA immediately after a damaging exercise demonstrated similar EIMD between groups. The absence of effect on EIMD might be due to the acute supplementation dose following exercise and is insufficient to change muscle phospholipid content relative to the 30 days of supplementation used in mentioned studies (DiLorenzo et al., 2014; Lenn et al., 2002).

In addition, subgroup analysis indicated that omega 3 consumption had a significant decrease in CK concentration in RCTs that had before and after exercise supplementation. In view of the above, the main feature of EIMD is skeletal muscle fibers disruption, especially the basal lamina sheath. About mechanical stimuli, particularly anaerobic training, previous studies suggested that it can increase muscle fibers microdamage imposed via contractions and based on the length, volume, and intensity; the damage level and muscle soreness may be persist chronically (Jäger et al., 2019; Nakhostin‐Roohi et al., 2016). Because of these mechanisms, omega 3 can affect EIMD more efficiently with before and after exercise supplementation protocol and for several days' consumption.

The strength of the present study is that it is the first meta‐analysis, which shows the omega 3 supplementation effect on indirect blood markers of EIMD. In addition, the main strength of our meta‐analysis is that we considered all published RCTs performed on the omega 3 supplementation effect on indirect blood markers of EIMD concentration, considering subgroup based on follow‐up times measurements after exercise (immediately, 24, 48, 72, and 96 hr after exercise). This meta‐analysis may provide baseline data and can guide other researchers to design new studies.

The high heterogeneity stated is the major limitation in this meta‐analysis. Many factors can affect this heterogeneity. Sex had a significant influence in other articles in serum CK activity (Stupka et al., 2000), and we mentioned the small numbers of women in the studies. In addition, due to unspecified dose of EPA and DHA in some studies, we could not perform subgroup analysis based on omega 3 dosages. Also, some participants can be less or more sensitive to changes in myocyte membrane permeability or may have dissimilar indices clearance rates because of different responses to training status (Morris, 2017; Rahimi et al., 2017). Therefore, the outcomes in this meta‐analysis are not reliable overall.

## 5 CONCLUSION

Briefly, the outcomes within the current meta‐analysis indicate that omega 3 supplementation is effective for alleviating EIMD that happens after exercise muscle damage. Further studies with diverse dosages of omega 3 and different exercise protocols are required to assess the best dosage and repetition per day for optimized recovery. In addition, in the present meta‐analysis, we focused on indirect blood markers including CK, LDH, and Mb levels, which associated with EIMD. Other items related to EIMD, such as inflammation markers, strength, and muscular function, have been prove to decrease after exercise. Other studies may be required to assess these outcomes after different exercise protocols and different dosages of EPA and DHA.

## CONFLICT OF INTEREST

Authors have no conflict of interest to declare.

## Data Availability

The data that support the findings of this study are available from the corresponding author, upon reasonable request.
